# From privatization to health system strengthening: how different International Monetary Fund (IMF) and World Bank policies impact health in developing countries

**DOI:** 10.1186/s42506-019-0013-x

**Published:** 2019-02-21

**Authors:** Saeed Sobhani

**Affiliations:** Department of Public Health Leadership, The Gillings School of Global Public Health, University of North Carolina at Chapel Hill, 470 Rosena Hall, CB, #7400, 135 Dauer drive, Chapel Hill, NC 27599-7400 USA

## Abstract

**Background:**

In the past several decades, the World Bank (WB) and the International Monetary Fund (IMF) have transitioned priorities from rebuilding European and Asian countries to decreasing poverty in developing countries. In addition, they evolved to be the world’s main financial sponsor to healthcare-related projects. Policies of these organizations forced some structural adjustment policies on many developing countries that resulted in negative consequences. This piece examines the impact of the changing policies of WB and IMF on the health of vulnerable populations and suggests potential recommendations for future improvements.

**Findings:**

Privatization of health services has become common around the world especially in the developing countries. Several reports documented negative effects of privatization on healthcare systems and vulnerable populations. Countries that received loans from the WB to privatize its social security system, for example Mexico, had drastic changes in the conditions of health. Public sector organizations faced budget reductions that led to an erosion of healthcare services, increased rates of unemployment, and lack of insurance of low-income people. The IMF’s monetarist strategies towards prioritizing fiscal restraint (low budget deficits) and price stability (low inflation) negatively impacted low-income people and increased the inequity in access to health care.

The present policies of these two organizations that focus on health system strengthening (HSS), and specifically incorporating existing *vertical* programs into health systems, had some critics. These programs, though many were successful, might result in adverse unintended effects for the health system and non-targeted populations. Some of the developing countries are unable to implement HSS projects properly.

**Conclusions:**

Both the past and the present policies of the WB and the IMF had adverse consequences on the health systems in developing countries. These policies should be re-considered. Developing countries should implement strategies to increase quality of care and improve equity in access to healthcare. Time-limited vertical programs should be developed carefully to avoid adverse unintended effects for the health system and non-targeted populations. Strategies at both operational and strategic levels to improve relations between the vertical and horizontal basics of the system should be adopted.

The World Bank (WB) was created in 1944 at the end of World War II, along the International Monetary Fund (IMF), to fund reconstruction efforts in many European and Asian countries [1]. In the last several decades, both organizations have transitioned priorities from rebuilding European and Asian countries to decrease poverty in developing countries [2]. In addition, they evolved from having a limited presence in global health to being the world’s main financial sponsor to healthcare-related projects [1, 2]. Policies of these organizations related to health systems initially emphasized macroeconomic, privatization, stability, trade liberalization, and public sector contraction [1–3]. However, along with these policies, they forced some structural adjustment policies on many developing countries that resulted in negative consequences [4]. IMF and WB policies began to shift as they recognized that open markets and economic management were insufficient; rather, efficient governance and powerful institutions are critical to alleviating poverty and addressing the health needs of a country [1–4]. Currently, one of the main focuses of these two organizations is on health system strengthening (HSS), and specifically incorporating existing vertical programs into health systems [1, 5]. This piece examines the impact that changing WB and IMF policies has had on the health of vulnerable populations and suggests potential recommendations for future improvements.

Privatization of health services has become common around the world especially in the developing countries as the World Bank’s adjustment health policy changed from government dominance to mix of public-private healthcare reform and private dominance [1–3]. Features of privatization include promotion of competition among providers and separation of financing from healthcare delivery. Advocates of privatization argued that it promotes efficiency, stimulates political and economic competition, and decreases corruption of public management [1, 3, 6]. On the other hand, opponents of healthcare privatization believe that the privatization in countries with lack of transparency could led to the higher administrative costs, gender discrimination, and higher profits for financial and investment corporations [4, 7, 8].

The opponents of healthcare privatization backed their arguments by highlighting the negative effects of privatization on healthcare systems and vulnerable populations [4, 7]. Mexico was one of the many countries that received loans from the WB in 1997 to privatize its social security system which drastically changed the conditions of health [7]. As a result, public sector organizations faced budget reductions that led to an erosion of healthcare services and left millions of unemployed and low-income people without insurance [7–9]. IMF’s monetarist strategies towards prioritizing fiscal restraint (low budget deficits) and price stability (low inflation) were unreasonably restrictive. These policies have prevented many developing countries from improving long-term public health investment by decreasing the percentage of gross domestic product (GDP) allocated to the underlying public health infrastructures [4]. These policies negatively impacted low-income people and increased the inequity in access to health care. Such strict strategies resulted in thousands of deaths due to tuberculosis in Eastern Europe as public healthcare had been weakened [8, 10].

As a result of these failures, IMF and WB have gradually recognized the need for HSS in developing countries to support their disease-specific efforts [8]. Most of the HSS projects done by them were vertically funded, managed, delivered, and monitored [11–13]. Vertical programming is desirable for these projects because they want to focus on a specific demographic population, disease, or health issue; they need a rapid response for specific measurable outcome objectives [11–13]. In addition, they could work on dual reporting structure to national health authority and to donor/sponsor and plan to provide certain services when a highly skilled workforce is limited. Finally, because health activities occur parallel to normal primary care activities, these projects are promoted in areas of poverty, isolation, epidemic disease, and poor health.

The World Health Organization (WHO) [14] definition of HSS contains two main components: first, “the process of recognizing and implementing the changes in policy in a country’s health system such that the country could respond better to its health system challenges,” and second, “any array of strategies and initiatives that improves one or more of the functions of the health system.” HSS is a mean to improve national and local health system outcomes [14, 15].

Using vertical programs in the implementation of HSS projects is driven by the assumption that focusing on a few well-concentrated interventions is an efficient way to improve the outcome and time response of the existing resources [11, 14–16]. To best utilize vertical programs, policy-makers use two methods: first, time-limited vertical programs, and second, indefinite vertical programs [11, 17].

Time-limited vertical programs should be developed carefully. These programs should avoid adverse unintended effects for the health system and non-targeted populations. These strategies should also describe how the vertical programs could be used to strengthen health systems, especially in terms of primary health care. For indefinite programs, there are strategies at the both operational and strategic levels that could improve relations between the vertical and horizontal basics of the system [16]. At the structural level, developing countries could use shared regulatory arrangements with solid control over the intersection between vertical and horizontal programs [18]. A good example of this mechanism is immunization coverage under the Expanded Program on Immunization (EPI). Since the 1970s, impressive and well-regulated EPI were implemented in many developing countries such as Pakistan and Ghana to ensure that all children under 1-year-old receive a basic immunization regimen as required by the World Health Organization (WHO) [12]. Other structural mechanisms are using systems, which permit joint planning and scrutinizing the established health system. A good example of using these strategies is Nepal that by using joint planning they overcome their key health challenges such as low vaccination rate. These mechanisms also allow the joint management of funding as described in the United Nations Sustainable Development Agenda [19]. There are some mechanisms at the operational level as well. Delivering general health services by using vertical programs and collaboration between vertical and horizontal program managers are some examples [11, 19]. For instance, Iran used the combination of vertical and horizontal planning to address the malnutrition in its children [17].

Despite of all these considerations and mechanisms, many critics still believe that some of the developing countries are unable to implement HSS projects properly [8, 20]. Countries such as Pakistan have suffered from chronically underfunded public investment, and remaining in compliance with the conservative policy of the IMF (i.e., low inflation policies) is not feasible for them [21]. To get inflation down, IMF forces developing countries to raise interest rates, the policy which economists refer it to as the “sacrifice ratio policy.” Based on this policy, a part of GDP will be sacrificed in order to get inflation rates down [22]. With current IMF policies, developing countries face lower growth rates and less taxes collected that result in less public health expenditure and public health investment. On the other hand, some countries such as Kenya and Tanzania benefit from these policies and they could reduce their new HIV infection rates to 62,000 and 55,000, respectively [23]. Based on the aforementioned arguments, the following are some recommendations for both IMF/WB and the countries that want to follow IMF/WB policies:

1. The IMF should extend the temporary flexibility that introduced to its recent agreements. IMF recent agreements with developing countries suspend the unnecessarily restrictive rules such as the low inflation and low budget deficits policies and provide more flexible rules. Such flexibility allows more space for developing countries to generate and allocate more internal resources to address the shortage of healthcare workers, especially in countries with a high HIV burden.
2. The IMF should reconsider its tactic to fiscal deficit and inflation rate. IMF should let the governments to borrow and explore more options in terms of public spending and development strategies. Some of these options, as Raciborska suggested, were to define and adopt a global standard, promote tools for financial transparency, provide small-scale technical assistance to build national capacity, and encourage dissemination of evidence based financial interventions [24].
3. IMF policy makers should involve a wider range of stakeholders such as health ministries, civil society, and health worker associations. They should let the discussions on macroeconomic policies take place in joint environment with other economic and social issues. Attempts to adopt an open and reasonable process of appointment for the leaders of the related organizations, giving a stronger role to the Executive Boards to oversee the work of the IMF, and make a structure of demonstration that better reflects the stakes of all state members are some examples of those attempts that should be done to make IMF and WB more transparent and more accountable [25].
4. Developing countries should implement strategies not only to increase quality of care but also to improve equity in access to healthcare. A good example of this is sets of recommendations in the “Egypt roadmap” to achieve social justice in health [26]. Increase financial protection for disadvantaged groups by equitable revenue collection, pooling for equitable distribution, purchasing strategically, and using new and evidence-based indicators for scaling the socioeconomic status of population [27] are some of the feasible strategies to achieve this goal.
5. Finally, donor governments should retest the experimental basis for IMF macroeconomic policy advice and stop complying with the IMF as the gatekeeper for their decisions on aid. A good example of this is the Bill & Melinda Gates Foundation as a major contributor to global health, which does not necessarily follow IMF or WB recommendations and its influences on international health policy, and the design of global health programs and initiatives is profound [28].

## Abbreviations

EPI: Expanded Program on Immunization
HSS: Health system strengthening
IMF: International Monetary Fund
WB: World Bank
WHO: World Health Organization
